# Factors Associated with Intrapartum Cesarean Section in Women Submitted to Labor Induction

**DOI:** 10.1055/s-0039-1688966

**Published:** 2019-06

**Authors:** Glaucia Regina Pfützenreuter, Juliana Coutinho Cavalieri, Ana Paula de Oliveira Fragoso, Karine Souza Da Corregio, Paulo Fontoura Freitas, Alberto Trapani

**Affiliations:** 1 Hospital Regional de São José Homero de Miranda Gomes, São José, SC, Brazil; 2 Universidade Federal de Santa Catarina, Florianópolis, SC, Brazil

**Keywords:** labor, induced labor, cesarean section, parto, parto induzido, cesariana

## Abstract

**Objective** To evaluate the results of induced labor and to determine the main factors associated with intrapartum cesarean section after patients being submitted to this procedure at the Hospital Universitário of the Universidade Federal de Santa Catarina (HU/UFSC, in the Portuguese acronym), Florianópolis, state of Santa Catarina, Brazil.

**Methods** A retrospective cross-sectional study that included all the pregnancies that resulted in single-fetus births, whose gestational-age was > 22 weeks and that had been submitted to labor induction at the HU/UFSC in the period from 2013 to 2016.

**Results** During the proposed period, 1,491 pregnant women were submitted to the labor induction protocol. In 1,264 cases (84.8%), induction resulted in labor, with 830 (65.7%) progressing to vaginal delivery. Gestational age ≥ 41 + 0 weeks was the most common indication for induced labor (55.2%), and vaginal administration of misoprostol was the most commonly used method (72.0%). Among these pregnant women, the cesarean section rate was of 34.3%. Considering the cases of induction failure, the cesarean section rate rose to 44.3%. The factors associated with cesarean section were: previous history of cesarean delivery (PR [prevalence ratio] = 1.48; 95%CI [confidence interval]: 1.51–1.88), fetuses with intrauterine growth restriction (IUGR) (PR = 1.82; 95%CI: 1.32–2.19), Bishop score ≤ 6 (PR = 1.33; 95%CI: 1.01–1.82), and induction time either < 12 hours (PR = 1.44; 95%CI: 1.17–1.66) or > 36 hours (PR = 1.51; 95%CI 1.22–1.92) between the beginning of the induction and the birth.

**Conclusion** Labor induction was successful in most patients. In the cases in which the final outcome was a cesarean section, the most strongly associated factors were: previous history of cesarean delivery, presence of fetuses with IUGR, and either excessively short or excessively long periods of induction.

## Abstract (pt)

**Resumo:**

**Objetivo** Avaliar os resultados da indução de trabalho de parto e determinar os principais fatores associados à realização de cesarianas intraparto em pacientes do Hospital Universitário da Universidade Federal de Santa Catarina (HU-UFSC), Florianópolis, SC, Brasil.

**Métodos** Trata-se de um estudo epidemiológico transversal que incluiu todas as gestantes de feto único e com idade gestacional > 22 semanas, submetidas a indução de trabalho de parto no HU-UFSC no período de 2013 a 2016.

**Resultados** No período proposto, 1.491 gestantes foram submetidas ao protocolo de indução. Em 1.264 casos (84,8%), a indução resultou em trabalho de parto, com 830 (65,7%) progredindo para o parto vaginal. Gestação ≥ 41 + 0 semanas foi a causa mais comum de indicação de indução de trabalho de parto (55,2%), e misoprostol foi o método mais utilizado (72,0%). Nessas gestantes, o índice de cesariana foi de 34,3%. Considerando os casos de falha de indução, o índice de cesariana sobe para 44,3%. Os fatores associados às cesarianas foram: história prévia de cesárea (RP [razão de prevalência] = 1,48; IC [índice de confiança]: 95% 1,51–1,88), fetos com restrição de crescimento intrauterino (RP = 1,82; IC95%: 1,32–2,19), índice de Bishop ≤ 6 (RP = 1,33; IC95%: 1,01–1,82) e tempo de indução < 12 horas (RP = 1,44; IC95%: 1,17–1,66), ou > 36 horas (RP = 1,51; IC95%: 1,22–1,92) entre o início da indução e o parto.

**Conclusão** A indução de trabalho de parto foi bem-sucedida na maioria das pacientes. Naquelas em quem o desfecho final foi a cesariana, os fatores mais fortemente associados foram: história prévia de cesárea, presença de fetos com restrição de crescimento intrauterino, e tempos muito curtos ou muito longos de indução.

## Introduction

Labor induction is a relatively common procedure in obstetric practice, and it consists of artificially triggering effective uterine contractions before labor spontaneously commences in pregnant women of gestational age > 22 weeks. The onset of labor depends on a series of coordinated and synchronized processes, such as persistent uterine contractions, cervical maturation, and descent of the fetal presentation.^1^ ^2^ ^3^ ^4^

In recent years, labor induction has become more frequent, currently comprehending ∼ 20% of pregnancies. Labor induction is indicated when the benefits to the mother and/or to the fetus are greater than the maintenance of the gestation.^5^ ^6^

The American College of Gynecology and Obstetrics (ACOG) recommends as indication for labor induction: chorioamnionitis, fetal death, gestational hypertension, preeclampsia and/or eclampsia, rupture of the amniotic membranes, pregnancy of ≥ 41 + 0 weeks, certain maternal conditions (e.g., diabetes, renal disease, chronic hypertension, among others), and fetal impairment (e.g., intrauterine growth restriction [IUGR], isoimmunization, and oligodramnia). Labor induction can also be recommended due to logistical issues, such as risk of rapid labor, fetal malformation incompatible with extrauterine life, distance to the location of the hospital, or psychosocial issues.^7^

Pregnancy of ≥ 41 + 0 weeks is the most frequent indication for labor induction. On the other hand, the indication for induction of elective labor (for the sake of convenience) is becoming ever more frequent. In a meta-analysis including 7 studies, it was noted that the elective induction rate ranges from 0.95% to 10%, showing a progressive increase in almost all settings.^8^

Several methods have been described to promote both cervical maturation and labor itself. Among the most commonly used methods are the administration of prostaglandins (misoprostol and dinoprostone), oxytocin, and Foley probe, which either alone or in a combination thereof help in the process of uterine cervix maturation and stimulate labor.^2^ ^9^ ^10^ ^11^

The traditional method to assess the ripeness of the cervix prior to labor induction is the cervical scoring system described by Bishop, known as the Bishop score.^12^ ^13^ ^14^ ^15^

Historically, there is no well-accepted definition to characterize labor induction failure. On the other hand, Spong et al^16^ point out as the criterion for induction failure the inability to generate regular contractions and cervical alteration after 24 hours of oxytocin administration with artificial rupture of the membranes, when possible.

It is known that the purpose of labor induction is the interruption of pregnancy because it is considered safer for the pregnant woman and/or to the fetus than the maintenance of the pregnancy, and that vaginal birth is a better option than performing an elective cesarean.^5^

Brazil is experiencing an epidemic of cesarean sections, with ∼ 1.6 million cesarean sections performed each year. In the last decades, the national rate of cesarean operations has progressively increased, and cesarean section surgery has become the most common mode of birth in the country. The rate of cesarean sections in Brazil is of ∼ 56%.^17^ A significant difference is noticed between the public health services (40%) and the private health services (85%).^17^

Therefore, the present study aimed to evaluate the results of labor induction and to determine the main factors associated with intrapartum cesarean section in women who were submitted to this procedure and eventually progressed to the active phase of labor in a public university hospital in the southern region of Brazil.

## Methods

This is a cross-sectional epidemiological study that included all the women who delivered a single and living fetus after being submitted to labor induction between the years 2013 and 2016 at the Hospital Universitário of the Universidade Federal de Santa Catarina (HU/UFSC, in the Portuguese acronym), Florianópolis, state of Santa Catarina, Brazil. Data were collected from birth records, medical charts, and from the eletronic information system of the hospital.

Women who failed the induction method were excluded from the present study for a better evaluation of the outcomes. Fetal death was also excluded so that postinduction results could be compared with those of other studies.

As for the patients whose induction resulted in labor (whether vaginal delivery or cesarean section), the associations between the induction outcome and the following variables were tested: induction indication, induction method(s), maternal age, parity, gestational age, integrity of the amniotic membranes, total time of labor induction, and status of the cervix at the beginning of the induction procedure (Bishop score). Neonatal aspects, such as birthweight, 5-minute Apgar score, and any record of meconium amniotic fluid, were also related to the delivery route.

The Bishop score is an assessement of the position, of the consistency, of the effacement (shortening of the cervix), and of the dilatation of the maternal cervix, as well as of the station of the fetal presenting part. The maximum score is 13, and a score of at least 6 evolves to vaginal birth within 6 hours in 90% of the cases, whereas in women with a score < 6, the course of labor is unpredictable.^13^ ^14^ Some other studies have also considered a score ≥6 as favorable for labor induction.^15^ Although Bishop described his method as a means to prognose the success of labor induction in parous women with cephalic presentation, today the system is used for every induction of labor proposed.^13^

According to the labor induction protocol used at the HU-UFSC, in all of the pregnant women with favorable cervix (Bishop score > 6), intravenous oxytocin should be started with an infusion pump, in a dose-escalation scheme until reaching labor or the maximum dose of the drug (2 mIU/minute, increasing 2 mIU every 30 minutes, to the maximum of 40 mIU/minute).

When the cervix is considered unfavorable (Bishop score ≤6), the process is recommended to be performed using vaginal misoprostol according to the gestational age: term gestation, 25 µg every 4 hours; ≥ 30 weeks and < 37 weeks, 50 µg every 4 hours; and < 30 weeks, 100 µg every 6 hours, with a maximum dose of 8 tablets. When there was no response to the complete misoprostol regimen, intravenous oxytocin, initiated after 4 hours of the last dose of misoprostol, was indicated in the regimen described above.

In cases of previous cesarean section and unfavorable cervix, the method used was the Foley catheter, with intravenous oxytocin initiation either as soon as it was spontaneously ejected or 24 hours after its introduction.

Induction failure was considered either when the patient did not trigger effective contractions (3 contractions every 10 minutes) after the maximal oxytocin dose, or when there was no cervical maturation after maximal misoprostol or Foley catheter timeout, even after the administration of an intravenous oxytocin combination.

Pregnancy of ≥ 41 + 0 weeks was an indication for labor induction. Intrauterine growth restriction had a lower percentage (3.3%) as an indication for labor induction. It was defined according to the relation between birthweight and gestational age at the time of labor. The standardization method used at the HU/UFSC is the Hadlock chart.^18^ ^19^

The statistical analysis was performed using SPSS for Windows, Version 16.0 (SSPs Inc., Chicago, IL, USA). The prevalence ratio (PR) was used as a measure of relative risk (RR), and the 95% confidence interval (95%CI) was calculated. The chi-squared test at the 95% confidence level (α <0.05) was used to calculate the statistical significance of these associations.

Next, a multivariate analysis was performed with logistic regression, including all of the factors that were associated with cesarean section in the univariate analysis.

The present research was conducted in accordance with the resolution number 196/96 of the National Health Council for Research with Human Beings and was approved by the Ethics and Research Committee on Human Beings of the UFSC under the number 067/2008.

## Results

**Fig. 1:**
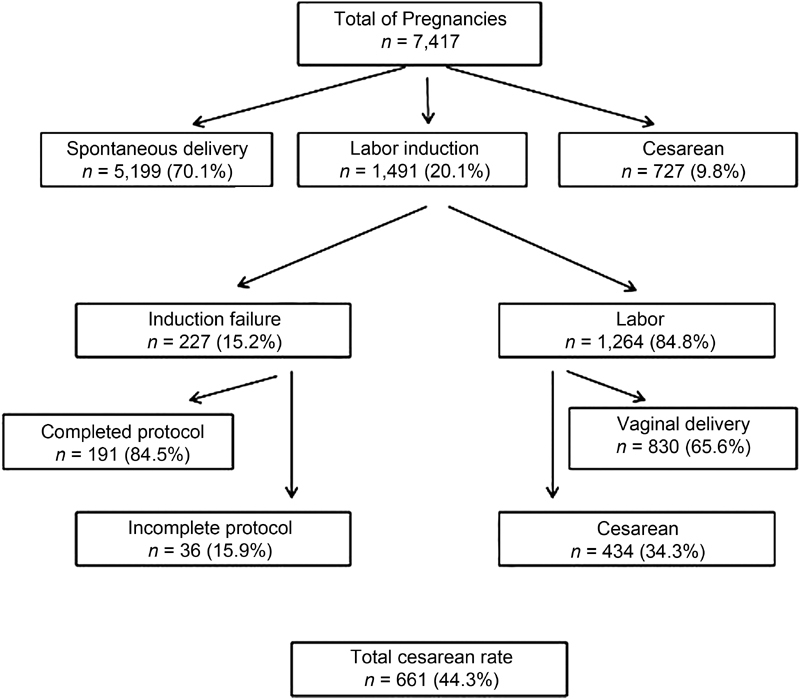
Flowchart of labor induction of the Hospital Universitário of the Universidade Federal de Santa Catarina, Florianópolis, state of Santa Catarina, Brazil, 2013 to 2016.

From January 2013 to December 2016, the HU-UFSC recorded a total of 7,417 single live births with gestational age > 22 weeks, and in 1,491 (20.1%) of the cases the patients had been submitted to the labor induction protocol. In 227 cases of induction (15.2%), the procedure failed. Within this group, the induction protocol was not completed in 36 cases (15.9%). In 1,264 cases (84.8%), the induction resulted in labor. Out of these cases, 830 (65.7%) progressed to vaginal delivery, and 434 (34.3%) required a cesarean section (Fig. 1). Considering all of the patients who underwent labor induction (labor + failures), the cesarean section rate was of 44.3%. In the induction failure group, there were 21 cases of pregnant women withdrawal due to incomplete protocol, 7 cases of fetal changes in cardiotocography tests, and 8 cases in which the cause of the suspension of induction was not addressed in the records of the patients. A total of 16% of the patients did not complete the protocol in the induction failure group (36 out of 227). This corresponds to only 2.4% of the total number of patients who underwent induction (36 out of 1,491).

The average age of the analyzed patients was 26.9 years old (standard deviation [SD] =  ± 6.5 years), and the average induction time was of 18.6 hours (SD =  ± 11.4). Pregnancy ≥ 41+ 0 weeks was the most common indication for labor induction (55.2%), followed by rupture of the amniotic membranes (11.9%), and gestational hypertension (10.8%). (Table 1).

**Table 1:** Outcome according to the main indication for labor induction

Labor Induction Indications	Vaginal delivery *n* (%)*	Cesarean section *n* (%)*	Total *n* (%)**	PR (95%CI)	*p-value*
Pregnancy of ≥ 41+ 0 weeks of gestation	475 (68.0%)	223 (32.0%)	698 (55.2%)	0.93 (0.81–1.06)	0.286
PROM	98 (64.9%)	53 (35.1%)	151 (11.9%)	1.02 (0.81–1.28)	0.851
Gestational hypertension	89 (65.4%)	47 (34.6%)	136 (10.8%)	1.01 (0.79–1.28)	0.999
Oligohydramnios	68 (59.1%)	47 (40.9%)	115 (9.1%)	1.19 (0.94–1.50)	0.142
GDM	65 (72.2%)	25 (27.8%)	90 (7.2%)	0.80 (0.57–1.13)	0.224
IUGR	13 (30.9%)	29 (69.1%)	42 (3.3%)	2.01 (1.62–2.49)	< 0.0001
Others	22 (68.7%)	10 (31.3%)	32 (2.5%)	0.91 (0.54–1.53)	0.722
Total	830 (65.7%)	434 (34.3%)	1264 (100%)		

Abbreviations: CI, confidence interval; GDM, gestational diabetes mellitus; IUGR, intrauterine growth restriction; PR, prevalence ratio; PROM, prelabor rupture of membranes.

*Percentage of type of delivery in relation to induction indication.

Administration of misoprostol was the mostly used method (72%), but there was no difference between the methods used and the way of delivery when association with oxytocin was tested (Table 2). Pregnant women with a history of previous vaginal delivery presented a higher rate of current vaginal delivery (79.6%) when compared with pregnant women who had not had any previous deliveries (57.2%; 450/787). On the other hand, pregnant women with a history of previous cesarean section had more intrapartum cesarean sections (56.6%) than those without this antecedent (30.3%; 323/1,168).

**Table 2:** Mode of delivery of women who underwent induction of labor and progressed to active labor

Type of induction	Vaginal delivery *n* (%)	Intrapartum cesarean section *n* (%)	Total *n* (%)	PR (95%CI)	*p-value*
Misoprostol	600 (65.9%)	310(34.1%)	910 (72.0%)	0.99 (0.88–1.11)	0.896
Ocytocin	145 (68.4%)	67 (31.6%)	212 (16.8%)	0.92 (0.74–1.13)	0.443
Foley Cateter	85 (59.8%)	57 (40.2%)	142 (11.2%)	1.16 (0.94–1.44)	0.154
**Total**	830 (65.7%)	434 (34.3%)	1,264 (100%)		

Abbreviations: CI, confidence interval; PR, prevalence ratio.

Regarding gestational age, post-term pregnant women (≥ 41 weeks) showed no increased risk of cesarean section (*p* = 0.09). Premature infants (< 37 weeks) presented a lower probability of cesarean section when compared with all of the other gestational ages (*p* = 0.005). When the time between the onset of induction and birth was between 12 and 24 hours, there were lower rates of cesarean sections (17.5%). On the other hand, a longer process (> 36 hours) was associated with an increase in the occurrence of cesarean sections (*p* < 0.0001) (Table 3).

**Table 3:** Maternal characteristics and induction length according to mode of delivery

Variables	Vaginal delivery (*n* = 830) n (%)	Cesarean section (*n* = 434) n (%)	PR (95%CI)	*p-value*
Maternal age				
< 20 years	143 (67.4)	66 (32.6)	0.91 (0.74–1.16)	0.442
20–35 years	560 (64.2)	307 (35.8)	1.03 (0.91–1.16)	0.608
> 35 years	127 (67.9)	61 (32.1)	0.95 (0.76–1.18)	0.614
Previous births				
Vaginal delivery	380 (79.6)	97 (20.4)	0.47 (0.39–0.57)	< 0.001
Cesarian sections	85 (43.4)	111 (56.6)	1.87 (1.60–2.18)	< 0.001
Gestational age				
< 37 weeks	82 (79.6)	21 (20.4)	0.57 (0.38–0.84)	0.005
≥ 41 weeks	449 (67.7)	214 (32.3)	0.87 (0.75–1.02)	0.090
PROM	89 (68.4)	41 (31.6)	0.91 (0.69–1.18)	0.486
Bishop score ≤6	743 (64.7)	405 (35.3)	1.41 (1.02–1.95)	0.026
Newborn weight				
< 2,500 g	57 (56.4)	44 (43.6)	1.30 (1.03–1.65)	0.042
> 4,000 g	106 (72.6)	40 (27.4)	0.77 (0.59–1.02)	0.073
Induction length				
< 12 hours	255 (55.1)	208 (44.9)	1.59 (1.37–1.84)	< 0.001
12–24 hours	351 (82.5)	74 (17.5)	0.40 (0.32–0.50)	< 0.001
24–36 hours	184 (63.7)	105 (36.3)	1.07 (0.90–1.28)	0.410
> 36 hours	40 (45.9)	47 (54.1)	1.64 (1.33–2.03)	< 0.001

Abbreviation: CI, confidence interval; PR, prevalence ratio; PROM, prelabor rupture of membranes.

There was no relationship between the delivery route and maternal age, amniotic membrane status, Apgar score, initial Bishop score, or the presence of meconium (Table 4). The presence of meconium was evaluated during labor after the rupture of the amniotic membranes or during the expulsive period.

**Table 4:** Distribution of the patients who responded to labor induction in relation to the neonatal outcomes and to the type of delivery

Variables	Vaginal delivery *n* (%)	Cesarian section *n* (%)	*p-value*
**Apgar score 5^th^ minute**			
< 7	6 (42.8)	8 (57.2)	0.081
≥ 7	824 (65.9)	426 (34.1)	
**Meconium**			
Yes	122 (60.7)	79 (39.3)	0.104
No	708 (66.6)	355 (33.4)	

In the multiple logistic regression analysis, the presence of fetus with IUGR, previous cesarean sections, Bishop score ≤ 6, or induction time of either < 12 hours or > 36 hours persists with significant association with cesarean sections (Table 5).

**Table 5:** Factors associated with cesarean section in patients who responded to induction and whose *p-value* was less than or equal to 0.05 in the initial bivariate analisys

Factors	Vaginal delivery (*n* = 830) *n* (%)	Cesarian section (*n* = 434) *n* (%)	APR (95%CI)	*p-value*
IUGR	13 (30.9%)	29 (69.1%)	1.82 (1.32–2.19)	< 0.0001
Previous Cesarian	85 (43.4)	111 (56.6)	1.48 (1.51–1.88)	0.003
Bishop score ≤ 6	743 (64.7)	405 (35.3)	1.33 (1.01–1.82)	0.029
Newborn weight < 2,500 g	57 (56.4)	44 (43.6)	1.10 (0.82–1.55)	0.072
Induction length				
< 12 hours	255 (55.1)	208 (44.9)	1.44 (1.17–1.66)	< 0.001
> 36 hours	40 (45.9)	47 (54.1)	1.51 (1.22–1.92)	< 0.001

Abbreviations: APR, Adjusted prevalence ratio; CI, confidence interval; IUGR, intrauterine growth restriction.

## Discussion

Labor induction is indicated when continuation of the pregnancy is thought to be associated with greater maternal or fetal risk than intervention to deliver the pregnancy, when there is no contraindication to vaginal birth.

Previous studies attempting to identify risk factors for cesarean delivery in patients undergoing labor induction have a more specific objective. Most of the times they analyzed only one variable, which makes it difficult to evaluate the implementation of a protocol. There are some studies that focus only on patients of term, some that focus only on patients with indication for induction, some that focus on a specific method of induction, and others that focus only on nulliparous and/or on multiparous patients.^20^ ^21^ ^22^ ^23^ ^24^ ^25^ ^26^ ^27^ ^28^ ^29^ ^30^ ^31^ ^32^ ^33^ In the present study, the overall intrapartum cesarean section rate was of 34.3%, which is slightly higher than those noted in other studies.^16^ ^31^ ^33^ The factors strongly associated with cesarean section were previous cesarean history, induction time > 36 hours, IUGR, and Bishop score ≤ 6.

The main purpose of labor induction is vaginal delivery, but it is well known that when labor is induced, the chances of vaginal delivery are lower than in spontaneous labor, especially in nulliparous women.^34^

As mentioned above, labor is induced in ∼ 20% of pregnancies and, in the United States, about one-third of deliveries occur by cesarean section.^4^ ^5^ Labor induction is employed to reduce the number of patients undergoing unnecessary cesarean sections. The United States has a high rate of cesarean sections when compared with other developed countries, and 90% of the patients who had a previous cesarean section undergo a new cesarean section. Given these data, several institutions seek to implement protocols to prevent the first unnecessary cesarean section, since this way of delivery has effects on the future reproductive life by increasing the risk of uterine rupture, as well as placental accretion, among others.^35^ ^36^ ^37^

According to Lappen et al,^35^ women with previous cesarean section had a higher risk of induction failure.^32^ It is important to note that the criteria of induction failure are not well defined in the literature and may vary according to the protocol of each institution; therefore, it is difficult to compare the results between studies.

Moreover, in induced labor, a history of previous cesarean section is a risk factor for a repeat cesarean section, which corroborates the data obtained in the present study.^35^ ^36^ ^37^ It is possible that patients who are undergoing labor induction and who had a previous cesarean section have a higher rate of intrapartum cesarean section that could be associated with individual obstetric characteristics.

Indication for labor induction for pregnancies of ≥ 41 + 0 weeks is an issue on which there is no consensus in the literature. In the multicenter ARRIVE trial, which evaluated the perinatal and maternal consequences of planned induction of labor at 39 + 0 to 39 + 4 weeks of gestation versus expectant management in > 6,100 low-risk nulliparous women across the United States, labor induction reduced the chances of cesarean delivery.^38^

Although elective induction at ≥39 weeks is a reasonable option for patients who want to schedule their delivery date, the differences in outcomes between elective induction and expectant management are small and do not warrant a strong recommendation for one approach versus the other.

Many authors point out that the longer the induction time, especially when the latent phase is prolonged, the greater the risk of induction failure.^25^ ^39^ ^40^ The definition of a “failed” induction of labor remains less certain when compared with the well-accepted standards for the diagnosis of arrested active-phase labor. One approach to diagnosing a failed induction is based on the duration of the latent phase. A study conducted by Grobman et al^41^ showed that labor inductions with latent phase > 15 hours had an increased risk of failure and a consequent progression to intrapartum cesarean sections.

This correlation corroborates the findings of the present study, which indicate that patients who had an induction time of > 36 hours had a cesarean section rate of 54.1%. For Spong et al,^16^ induced labors appear to take longer than what is traditionally estimated. However, further investigation is required to establish a standard minimum duration for the latent phase of a labor induction.

When comparing the indications for labor induction, it is possible to note that there was a higher cesarean rate in fetal indications than in maternal ones, which is in agreement with the study conducted by Parkes et al.^42^ These findings confirm the logical hypothesis that cesarean section is more common in the suspicion of fetal impairment.

The Apgar score is described in the literature as a factor unrelated to induction failure or to the risk of cesarean section.^42^ This statement is in agreement with the results obtained in the present study. However, during labor induction, fetal vitality is usually consistently analyzed, thus allowing the diagnosis of acute fetal distress at an early stage. Therefore, intermittent monitoring of fetal vitality is a viable alternative to avoid birth of newborns with a low Apgar score.^43^

Some studies report that IUGR is a protective factor against cesarean section.^18^ ^19^ ^27^ Nevertheless, in the present study, fetal growth restriction was a statistically significant risk factor for intrapartum cesarean section.

Although some studies report that gestational diabetes is a risk factor for cesarean section, only 27.8% of the diabetic patients induced were submitted to cesarean delivery.^25^ This low percentage can be explained by the probable exclusion of cases with macrosomia, in which patients undergo either elective cesarean section or spontaneous labor.

## Conclusion

In conclusion, since nowadays many pregnancies are reaching 41 weeks of gestation without going into spontaneous labor, it is important to further investigate the existing labor induction protocols. Therefore, to know of and to learn about the risks and benefits of this technique, it is necessary not only to review the protocols and to make any possibly necessary changes in the methods and approaches, but also to inform the patient (and her partner/companion) who will be submitted to the induction. The labor induction protocol used at the HU/UFSC resulted in an adequate response, in which the factors strongly associated with cesarean section were a previous history of cesarean birth, fetuses with IUGR, an induction time > 36 hours, and a Bishop score ≤ 6. Therefore, further efforts are important to lower the rates of a 1^st^ unnecessary cesarean section, as well as to better implement the existing protocols. The results of the present study reaffirm the concept of the effectiveness of the labor induction protocol used at the HU/UFSC. The present study was designed and its data were collected in a large hospital that follows the protocols of the main gynecology and obstetrics societies. The most important limitation of the present study is that different professionals assessed the Bishop scores and, therefore, there may be a significant variability in interpretations by examiners regarding the indication for the method of labor induction. The exclusion of patients who failed to respond to induction, and thus did not evolve into labor, also defines the limitations of the present study. However, this exclusion allowed a comparative analysis of the intrapartum cesarean section rate in patients who actually went into labor by means of induction.
